# When the Helmet Is Not Enough: Forensic Multidisciplinary Reconstruction of a Deadly Motorcycle Accident

**DOI:** 10.3390/diagnostics12102465

**Published:** 2022-10-12

**Authors:** Antonio Maria Catena, Michele Treglia, Luigi Tonino Marsella, Marcello Locatelli, Enrica Rosato, Abuzar Kabir, Martina Bonelli, Cristian D’Ovidio

**Affiliations:** 1 Institute of Legal Medicine, University of Rome 2 “*Tor Vergata*”, 00133 Rome, Italy; 2 Department of Pharmacy, University of Chieti-Pescara “*G. d’Annunzio*”, Via dei Vestini 31, 66100 Chieti, Italy; 3 International Forensic Research Institute, Department of Chemistry and Biochemistry, Florida International University, Miami, FL 33199, USA; 4 Department of Legal Medicine, University of Chieti-Pescara “*G. d’Annunzio*”; 66100 Chieti, Italy

**Keywords:** accident, trauma, brain injury, forensic pathology, autopsy, reconstruction of trauma, multidisciplinary profiles

## Abstract

We report the case of a 54-year-old man who died in a motorcycle accident due to loss of control of the vehicle on a viaduct. No other vehicles were apparently involved, except for a car hit by the motorcycle after it fell. A post-mortem CT scan (computed tomography scan) was performed showing complex head trauma with a subarachnoid hemorrhage and multiple skull and facial bone fractures. A forensic cinematic reconstruction performed by an engineer was needed to exclude other incident causes other than the loss of control. The multidisciplinary approach that included autopsy findings, a cinematic reconstruction, a helmet test and an examination played a key role in clarifying the dynamics of the accident, allowing us to explain how the death occurred despite the motorcyclist’s helmet use. The cause of death was identified as a penetrating head trauma with cerebral material exposure, produced by the impact of the head against a fixed bolt in the guardrail base. Despite the use of the helmet, the impact force was enough to render the protection ineffective and allowed the bolt to penetrate through the helmet and the skull.

## 1. Introduction

It is well known that motorcyclists involved in accidents have a high risk of severe trauma due to the lack of protection of the motorbikes [1]. Although lesions are reported to affect the entire body, mostly the head and thoracic areas [2,3,4], head trauma still represents one of the major causes of death among riders [3,5]. This is because the head is particularly vulnerable and exposed to trauma in motorcycle crashes, even if it is protected by a helmet. In recent decades, considerable effort has been put into reducing the risk of fatal head injuries among motorcyclists, especially through laws on road safety and making helmet usage mandatory [6,7,8]. These laws, as broadly reported, effectively reduced the occurrence of fatal crashes thanks to the protective potential of helmets [9,10,11], decreasing mortality by a solid 42% when properly used [8].

Despite these laws, there are still many risk factors and incorrect behaviors that can lead to very serious accidents which turn out to be fatal in spite of the helmet. Major risk factors may be behavioral, like speeding, risky driving, alcohol and drugs abuse, geophysical, such as road geography, weather and surface conditions, and situational, for example traffic and objects on the site of impact and many others more [1,12,13,14,15]. All these factors can contribute to making the helmet’s protection ineffective in case of accidents [14], not only by causing polytraumas, but also overmatching impact resistance of the helmet itself when the head represents the primary impact site. These circumstances may lead to unclear situations creating difficultes in the assessment of the causes and the dynamics of death, like in the case herein reported.

The multidisciplinary approach reported herein was crucial to assess the exact death dynamic. Data collected by pathologists and licensed medicine doctors during the autopsy and histological examinations, along with toxicological and radiological findings, were integrated with the dynamic reconstructions and the helmet and guardrail appraisal, carried out by the engineers, making possible a solid explanation of the event during the preliminary stages of the trial.

## 2. Case Presentation

A call to the Police Department of Pescara (Italy) reported a motorcycle crash with a victim on a suburban viaduct approaching the city area. Apparently, according to the witness statements, no other vehicles were directly involved in the accident, except for a car struck by the motorcycle at the end of its slipping. The victim was a 54-year-old man who was riding a large displacement bike on his way to work.

**Figure 1:**
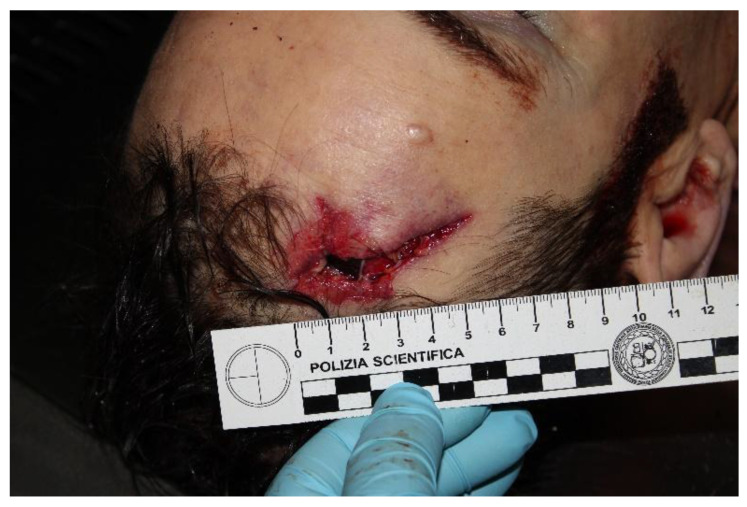
*“Bullet-like”* lesion on right frontotemporal area of the victim’s head.

The victim presented with a right frontotemporal-penetrating *“bullet-like”* lesion with a partial cerebral herniation (Figure 1).

**Figure 2:**
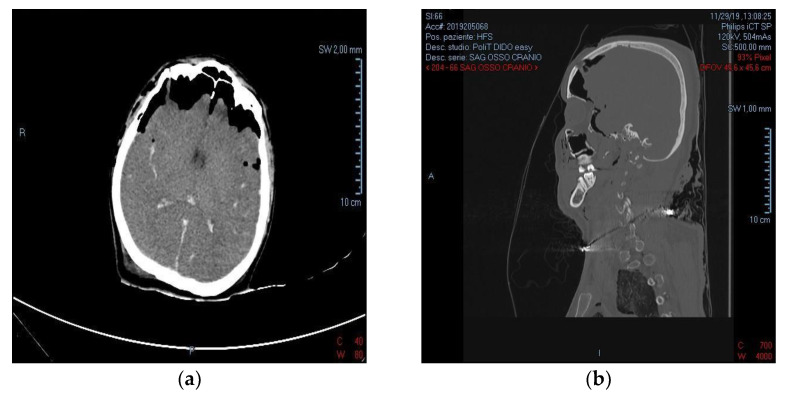
CT scan frames of the victim. (**a**) Transverse plane showing pneumocephalus and hemorrhages; (**b**) Sagittal plane showing the penetrating lesion of the skull.

Before the necroscopic assessment a CT scan was performed. It revealed multiple fractures of facial bones (zygomatic, nasal and orbital fractures) and, most importantly, showed large fractures of the right frontal and temporal bones with widespread signs of subarachnoid hemorrhage (SAH), bilateral intraventricular hemorrhage (IVH) and pneumocephalus (Figure 2).

**Figure 3:**
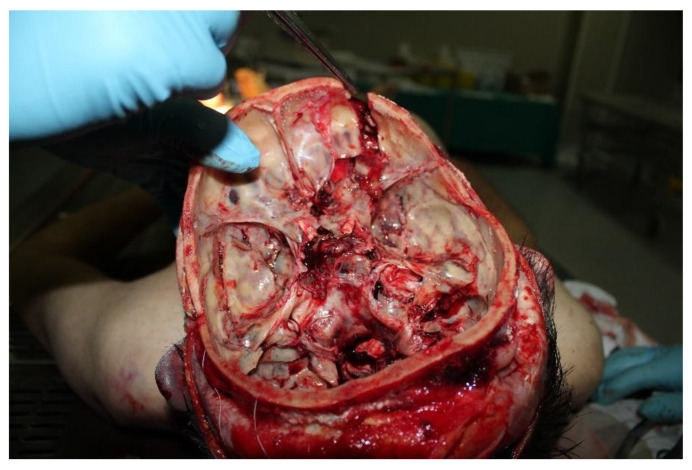
Details of the cranial base fracture produced by the impact.

The autopsy allowed the detection of multiple internal organ contusions and a sternum fracture consequent to thoracic trauma, involving both lungs, the aortic arch, and the first tract of the descending aorta. The dissection of the skull confirmed the CT results and made possible a better assessment of the grade of the cranial injuries. The presence of SAH and IVH was ascertained and furthermore, it was found that the fracture rim, produced by the penetrating trauma, deepened from the hole through the cranial base, causing a separation between the anterior and the middle cranial fossae (Figure 3). The cause of death was identified as a penetrating head trauma with cerebral material exposure.

**Figure 4:**
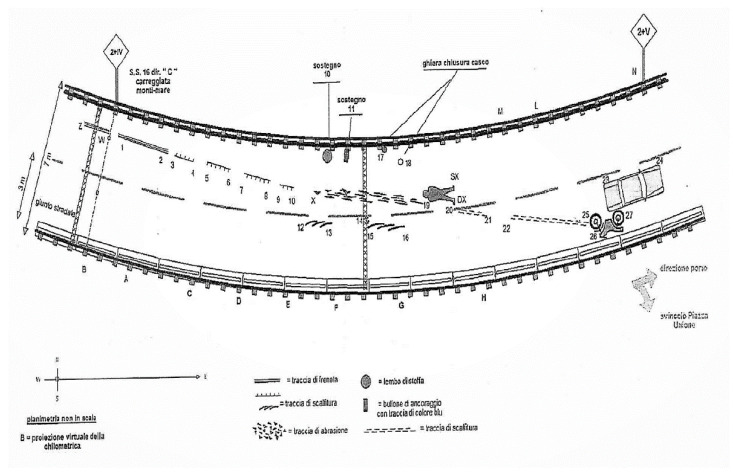
Simplified scheme representing dynamic reconstruction of the accident.

**Figure 5:**
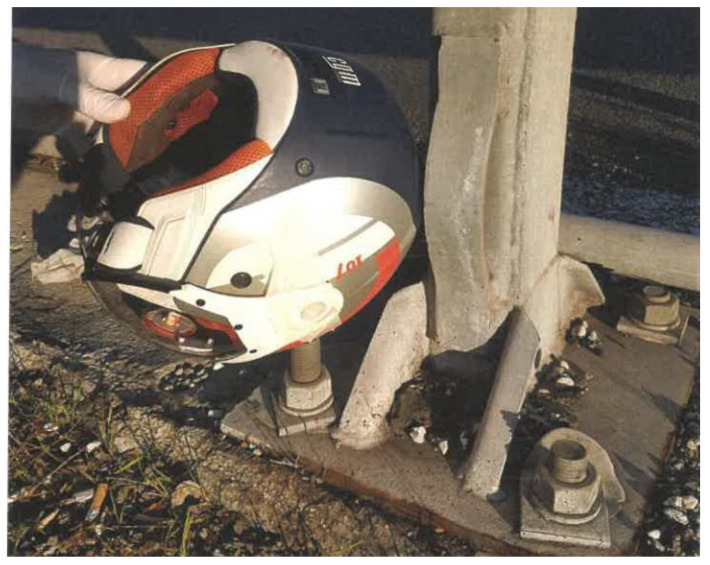
Biker’s helmet positioned on the impact site by the engineer during the site inspection.

The toxicological tests yielded negative results, as no traces of alcohol or drugs were detected at any levels in the victim’s biological samples. Based on the cinematic reconstructions, the biker was driving at high speed, at least 93 km/h with the speed limit set at 50 km/h, in the fast lane approaching a mild left turn on the viaduct. The victim tried to brake for the traffic jam ahead and suddenly lost control of the motorcycle. The rider fell on his left side, hitting the road surface near the guardrail, on the inside part of the curve, and then rolled through the center of the lane for a few meters (Figure 4). The appraisal of the open-face helmet (OFH) worn by the victim showed that it was homologated in respect to the circulation standard. Furthermore, the inspection assessed the compatibility between the hole on the helmet and the impact against a fixed bolt on the guardrail base (Figure 5). Despite the use of the helmet, the impact energy (about 1130 Joules) was enough to render the protection ineffective and allowed the bolt to penetrate through the helmet.

## 3. Discussion

As previously mentioned, the protective effect of the helmet and its capability to reduce the risk of severe head trauma and mortality in motorcyclists has been widely recognized by numerous studies [6,7,8,9,10,11,16,17,18]. In fact, when comparing the two groups, helmeted vs non-helmeted riders, the former group has less risk of suffering traumatic brain injuries (TBIs), cerebral hemorrhages and skull fractures [9,10,11,16,17,18], due to the protective effect of the helmet. Furthermore, fatal outcomes in motorbike crashes where a helmet was not used are much higher compared to crashes where a helmet was worn [9,11,16,17,18]. Going deeper into the matter, several studies have shown how different helmet types offer different protection levels against head, neck and face injuries [19,20,21]. Full-face helmets (FFHs) proved to be the safest, being able to reduce not only TBIs but also face traumas. In comparison, half helmets have the lowest protective potential, while open-face helmets (OFH) are placed between the previous two types, providing a fair amount of protection from TBIs but lacking face protection. So, helmets affect the injury types along with different kinds of impact dynamics [22].

In terms of lesions, it could be said that a certain type of accident corresponds to a certain set of injuries, being able to identify them as “typical” based on the data previously mentioned [19,20,21,22]. Nevertheless, because of the large number of variables playing a role in the complex dynamics of a crash, there are some cases where “atypical” injuries can be observed. In these situations, it is very important never to underestimate any single factor. This is particularly true in the forensic context, where every detail can make a difference in the establishment of the causes and dynamics of death. These details can completely change the plausibility, solidity and truthfulness of the evidence during the trials, even affecting their final outcomes.

Therefore, during forensic activity it is of the utmost importance to provide as much information as possible to explain how and why the death occurred. The conclu-sions must be consistent with the objective data, collected from a wide variety of sources including medical data from first responders, hospitals and autopsy, but also with the circumstantial and technical data from witnesses, police reports and the site inspection [14]. The elaboration of such a large amount of information may require other types of expertise, not only medical but also, and above all, technical. For this reason, forensic pathologists can benefit from collaboration with different professional figures.

In the case reported, the cooperation with engineers was crucial not only to clarify the exact incident dynamics but also to explain why and how the helmet protection was not sufficient to resist the impact causing the motorcyclist death. This was possible by integrating the kinematics data with the data from the helmet appraisal and the autopsy findings.

**Figure 6:**
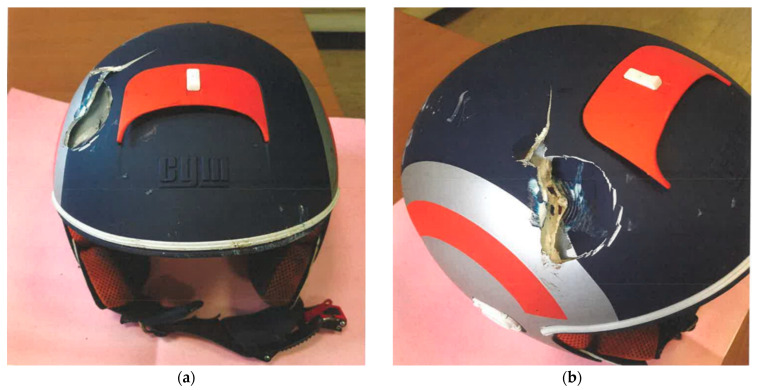
Biker’s helmet images taken during appraisal. (**a**) Frontal view of the helmet; (**b**) Right view showing the damage caused by the impact against the bolt.

The kinematic reconstruction assessed that there was a loss of control of the bike, which is one of the most common risk factors of fatal motorbike accidents [15]. The left side of the motorcycle slipping without other vehicles involved in the fall would have certainly procured bruises, abrasions and probably some fractures, which were in fact present on the corpse, but would have minimal involvement of the head. Indeed, a pre-emptive examination of the helmet allowed investigators to exclude a high-energy impact of the left side of the head on the ground, since only abrasion marks and minor dents were present on this side (Figure 6).

Indeed, the left portion of the head, as the CT scan and the autopsy revealed, was barely affected compared with the right portion. On the cadaver, only minor bruises and small linear fractures of the zygomatic arch and external orbital wall were found on the left side as a result of the first impact against the ground. On the other hand, the right portion of the head presented a frontotemporal-penetrating “bullet-like” lesion with partial cerebral herniation. From the aforementioned lesion, a large fracture rim deepened from the hole to the cranial base, involving the parietal and frontal right bones, along with the sphenoid, nasal bones and right orbit, causing a separation between the anterior and middle cranial fossae and lower part of the left temporal bone. Another fracture rim originated from the penetrating lesion, proceeding backwards and involving the right temporal bone. At the soft-tissue level, the direct contusion and laceration of right frontal cerebral lobe and occipital lobes contrecoup contusion with SAH and bilateral IVH were detected. Although there was the presence of diastasis between the anterior and middle cranial fossae, no damage involving the Willis circle or optic chiasm was reported. Moreover, no major lesions were found in the thoraco-abdominal district that could have represented an immediate life-threatening situation; in fact, the contusions involving both lungs, aortic arch and the first tract of the descending aorta were not so severe to hypothesize a major involvement in the cause of death.

Through the exclusion of other death causes, made possible by the accurate autopsy and the histological and toxicological findings, as expected, the penetrating lesion in the right frontotemporal area, which perfectly matched the lesion in the right half of the helmet, turned out to be the lethal injury that led the biker to death almost instantly.

As is well known, traumatic brain injury can be produced by direct and indirect mechanisms, leading to primary and secondary injuries with focal and diffuse damage that can both be present at the same time. Primary injuries, as consequences of trauma, result in fractures, epidural and subdural hematoma, contusions, focal microvascular and axonal injuries. These lesions, as result of complex biochemical pathways, lead to secondary injuries, consisting of diffuse ischemic damage by alterations of cerebral circulation and metabolism, swelling and augmented intracranial pressure (ICP), which are the basis of diffuse damage that can occur hours/days from the primary in-jury [23]. By raising the ICP, death can occur from the compression and herniation of the brainstem.

In the case reported, primary damage was clearly evident; however, except for the laceration, its presence was moderate and circumscribed. Autopsy and histological analysis did not show any signs of secondary damage. According to the reported data, it was concluded that the death occurred almost instantly via a mechanism similar to a bullet injury. In fact, the penetrating trauma led to a laceration of the cerebral pa-renchyma and to a pneumocephalus, with moderate SAH and IVH. Consequently, the augmented ICP allowed the external herniation of a frontal right lobe portion and the partial herniation and compression of the brainstem, resulting in cardio-respiratory arrest due to brain herniation.

The crash site inspection, along with the kinematics data, made it possible to assess that the biker’s head collided with a fixed bolt of the guardrail base, which protruded much more than it should have, with the head absorbing the residual energy of about 1130 Joules, overtaking the endurance limit of the helmet. To better understand the situation, studies on the structural composition of various homologated helmets were evaluated, and further investigations on the helmet and guardrail structure were conducted. It was ascertained that the helmet homologation test was carried out by colliding a helmet sample at 27 km/h, 27.54 km/h and 35.32 km/h against both plain and wedge-shaped surfaces. Therefore, the helmet was homologated following “Reg. ECE/ONU 22/05” standards.

All the collected data made it possible to demonstrate, during the preliminary hearing, that even if the biker had hit a regular bolt the outcome would have been the same. So even a regular bolt, as demonstrated by the cinematic assessments performed by the engineers, would have ended up hitting the head anyway because of the helmet thickness, which was smaller than the minimum bolt protrusion, and because of the impact energy and angle. On the basis of all the medical investigations, it was possible to assess that the motorcyclist would have suffered potentially fatal injuries in any case, due to the enormous amount of energy concentrated on such a small area. The impact energy was capable of inflicting serious damage to the head and brain even without the penetrating trauma to the skull.

## 4. Conclusions

The multidisciplinary approach including medical and non-medical professionals has proved to be a valuable tool in forensic practice when the case requires experts in other fields. A simultaneous integrative data analysis by different professional figures can make a difference during trials when the causes and dynamics of death are not clear and particularly difficult to establish, giving plausibility, solidity and truthfulness to the results for presentation of evidence in court. The case reported herein is just an example of how this can be performed with good results, and we hope that it can be helpful to anyone who wants to study more in depth the practical and experimental implications of this kind of approach.

## Data Availability

Not applicable.
